# Emotion and anxiety potentiate the way attention alters visual appearance

**DOI:** 10.1038/s41598-018-23686-8

**Published:** 2018-04-12

**Authors:** Antoine Barbot, Marisa Carrasco

**Affiliations:** 1 Department of Psychology, New York University, NY New York, USA; 2 Center for Visual Science, University of Rochester, NY Rochester, USA; 3 Center for Neural Science, New York University, NY New York, USA

## Abstract

The ability to swiftly detect and prioritize the processing of relevant information around us is critical for the way we interact with our environment. Selective attention is a key mechanism that serves this purpose, improving performance in numerous visual tasks. Reflexively attending to sudden information helps detect impeding threat or danger, a possible reason why emotion modulates the way selective attention affects perception. For instance, the sudden appearance of a fearful face potentiates the effects of exogenous (involuntary, stimulus-driven) attention on performance. Internal states such as trait anxiety can also modulate the impact of attention on early visual processing. However, attention does not only improve performance; it also alters the way visual information appears to us, e.g. by enhancing perceived contrast. Here we show that emotion potentiates the effects of exogenous attention on both performance and perceived contrast. Moreover, we found that trait anxiety mediates these effects, with stronger influences of attention and emotion in anxious observers. Finally, changes in performance and appearance correlated with each other, likely reflecting common attentional modulations. Altogether, our findings show that emotion and anxiety interact with selective attention to truly alter how we see.

## Introduction

Humans do not perceive the visual world with high fidelity, but rather represent visual information throughout a series of highly interactive and dynamic visual processing stages. Each time we open our eyes, we are confronted with an overwhelming amount of information, far more than our brain can effectively process at any given time. Selective attention^1,2^ is one of the key mechanisms our brain relies on to manage its limited processing resources and represent our environment in a subjectively meaningful way. Spatial covert attention^1,2^ allows us to select and prioritize the processing of visual information at relevant locations of our visual field without shifting gaze, helping signal relevant events and decide where to look next. Sudden changes in our environment, such as abrupt and salient object onsets, constitute spatial visual cues that result in the reflexive and automatic orienting of spatial attention, known as exogenous attention^1^. Exogenous covert attention has been shown to modulate early neural processing and improve performance in many visual tasks^1–4^. The mere presence of an exogenous cue can affect the response to subsequent stimuli presented near the cue’s location, even when attentional cues are known to be irrelevant for the task at hand and are not perceived consciously. The effects of exogenous attention peak ~100–120 ms after cue onset and decay quickly thereafter. This transient and reflexive attentional orienting plays a major role in our capacity to efficiently interact with our environment.

Emotional signals present in our environment can interact with attentional selection, facilitating the detection and adaptive responses to salient events and potential threats^5–8^. For instance, affective information has been shown to confer an advantage in many visual tasks^5,7–11^, such as visual search and visual discrimination. By modulating attentional mechanisms, responses to external emotional information can indirectly affect how we see. The amygdala is a brain structure that plays a vital role in signaling impending threats and possesses direct feedback projections to brain regions involved in attentional control as well as to cortical visual areas, including V1^6,12^. Physiologically, threat-related signals, such as fearful faces, have been shown to activate the amygdala and modulate activity in cortical visual areas^5,7,12,13^. At the behavioral level, modulatory signals in response to threats are assumed to underlie the findings that fearful faces can modulate the effects of selective attention and affect early visual processing^7–11,14–16^. The emotional valence of attentional face cues benefits contrast sensitivity performance by modulating the attention field size via feedback from the dorsolateral prefrontal cortex (DLPFC) to V1^17^, consistent with the normalization model of attention^3^. Besides the influence of external emotional stimuli on exogenous attention and perception, internal states such as arousal and anxiety can shape ongoing neural activity and interact with the attentional selection of incoming sensory inputs^7,10,18–22^. For instance, anxiety is associated with augmented amygdala activity and reduced prefrontal attentional control^18–20^, which could explain why high trait-anxiety participants exhibit stronger attentional effects on performance in response to emotional cues^9,10,21,22^.

Over the past decade, several studies have shown that exogenous covert attention not only improves performance, but can also alter the appearance of visual information around us^23–29^. Here, we asked whether the influence of emotional cues on exogenous attention not only affects perceptual performance, but can also impact our perception so deeply that it actually alters how visual information subjectively appears to us. In addition, we investigated whether individual trait-anxiety modulates the effects of attention, as well as the way emotional signals interact with attention, to alter how we see.

## Results

**Figure 1:**
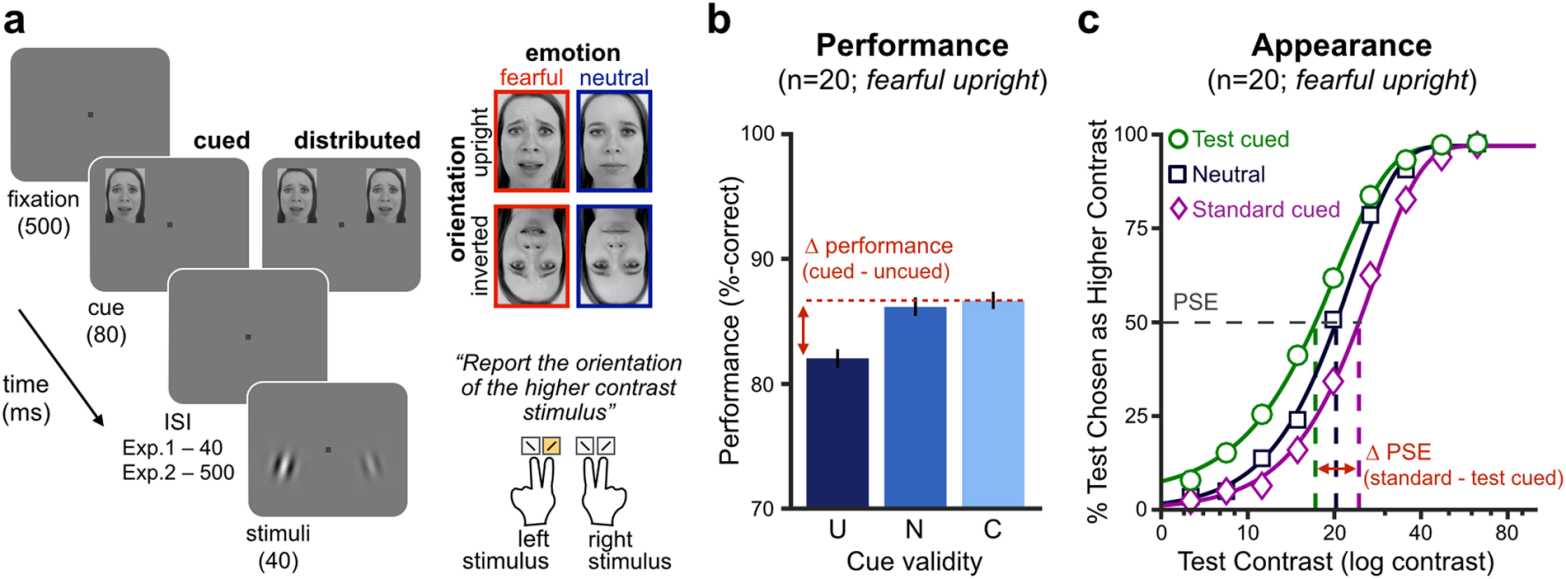
(**a**) Trial sequence. In each trial, observers reported the orientation of the higher contrast stimulus, while one (cued) or two (distributed attention) non-informative precues were used to manipulate spatial attention. A set of 11 Eckman face stimuli with either fearful or neutral emotional valence, and presented either upright or inverted, served as attentional precues (as in previous studies^10,11^; example face stimuli only used for illustration purpose). The inter-stimulus interval (ISI) between cue offset and stimuli onset was either optimal for exogenous attention (Exp. 1) or lengthened to prevent the transient effects of exogenous attention and rule out response bias (Exp. 2). (**b**) Attentional cueing effects on performance (here for fearful upright cues in Exp. 1) were computed for each observer by taking the difference in performance between the cued and uncued conditions (U: uncued; N: neutral; C: cued). Error bars represent ±1 SEM. (**c**) Attentional cueing effects on perceived contrast (here for fearful upright cues in Exp. 1). Each curve represents the Weibull fit for each cueing condition (test cued, standard cued, or neutral) of the probability to report the test stimulus as being higher in contrast than the standard stimulus (20% reference contrast) as a function of test contrast. Vertical dashed lines represent point-of-subjective equality (PSE) estimates of the test contrast at which both stimuli were equally likely to be reported as higher in contrast. Attentional effects on perceived contrast were computed for each observer by taking the difference in PSE between the standard-cued and test-cued conditions.

Observers (n = 20) saw two oriented Gabor stimuli (2 cycle/deg) presented simultaneously at isoeccentric (8 deg) locations to the left and right of central fixation (Fig. 1a). One stimulus had a reference contrast (20%; standard stimulus) while the other varied in contrast (6.3–63%; test stimulus). Observers were asked to report the orientation (CCW or CW) of the higher contrast stimulus, allowing us to measure both orientation discrimination performance of the chosen Gabor and perceived contrast with one button press^23–27^. In Experiment 1, spatial attention was manipulated by presenting non-informative peripheral precues 120 ms before stimulus onset, either at one location–focal attention condition–or at both locations–distributed attention condition. Precues were upright or inverted Ekman faces with either neutral or fearful expression (Fig. 1a), which have been successfully used to manipulate exogenous attention^10,11,15^. Upright fearful faces have been shown to result in stronger attentional effects relative to neutral-emotion precues. This preferential effect towards fearful faces disappears when faces are inverted^11,14,30^, consistent with disrupted emotional processing, and ruling out low-level visual differences between fearful and neutral face cues as responsible for the effect of emotion on attention.

For each of the four precue conditions (i.e, upright/inverted x fearful/neutral-emotion), we separately computed the effects of attention on orientation discrimination performance and on perceived contrast. The effects of attention on orientation discrimination were computed for each precue condition by measuring the differences in performance when the reported stimulus was cued minus uncued (Fig. 1b). We assessed the effects of attention on perceived contrast by computing the difference in the point-of-subjective equality (PSE; i.e. the test contrast level required to perceive both stimuli as having equal contrast) between the standard-cued condition and the test-cued condition (Fig. 1c). As illustrated in Fig. 1c, in the neutral attention condition, the PSE is at physical equality (i.e., the test contrast matches the 20% standard contrast). Enhanced perceived contrast with attention results in a lower PSE when the test location is cued, as less contrast is needed for the test stimulus to match the standard stimulus. Conversely, when the standard location is cued, the standard stimulus appears more contrasted and a higher test contrast is needed for both stimuli to appear subjectively indistinguishable. Trait anxiety was assessed for each participant using the State-Trait Anxiety Inventory^31^ (STAI). We then analyzed how the emotional valence (fearful *versus* neutral) and face orientation (upright *versus* inverted) of the attentional precues modulated the effects of attention as a function of the observer’s trait anxiety.

Here we show for the first time that emotional cues not only boost the effects of attention on performance, but also potentiate the way attention enhances contrast appearance. Moreover, our results reveal that both the effects of attention and the emotional potentiation of attention are mediated by the observer’s trait anxiety level.

**Figure 2:**
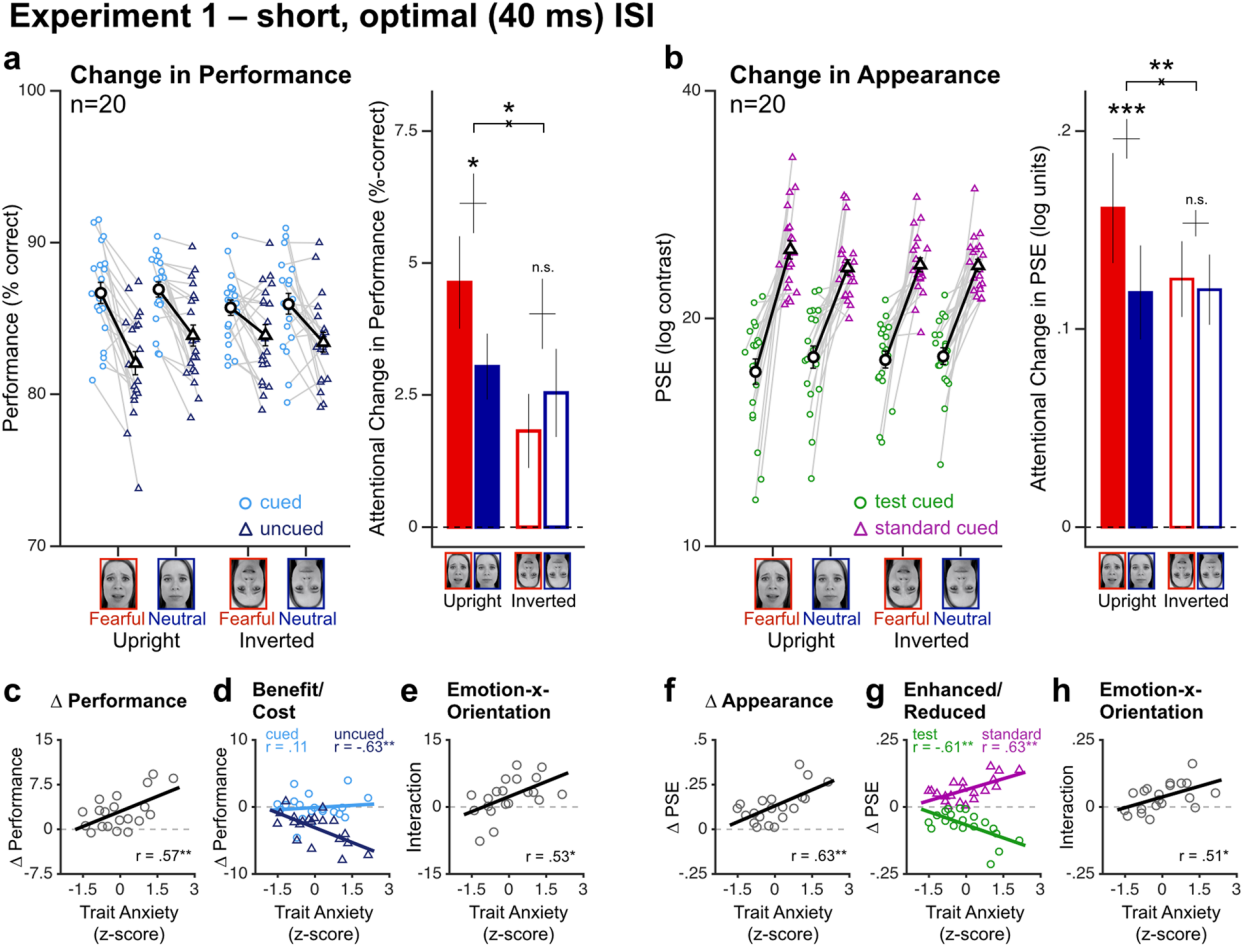
Experiment 1 (short ISI, optimal for exogenous attention). (**a**,**b**) Attention improved orientation discrimination performance (**a**) and enhanced perceived contrast (**b**) in all of the four different precue conditions. Each colored symbol represents an individual observer in each of the 4 different cue types (light blue/green circles: cued performance/test-cued PSE; dark blue/purple triangles: uncued performance/standard-cued PSE). Larger black symbols represent group-averaged values, with ±1 SEM error bars. Consistent with the idea that emotion potentiates the effects of attention, attentional effects were larger for fearful than neutral-emotion precues, but only when presented upright. (**c**,**d**) Individual trait anxiety correlated with the overall changes in performance, with larger attentional costs in high-anxiety observers. (**f**,**g**) Increased trait anxiety was also associated with larger attentional enhancement in perceived contrast. (**e**,**h**) The emotional potentiation of attention correlated with trait anxiety, with stronger interaction effects on performance (**e**) and perceived contrast (**h**) in high-anxiety observers. *: *p < 0.05*; **: *p < 0.01*; ***: *p < 0.001*.

Consistent with numerous studies^1,3,4^, attention improved performance regardless of the type of face cues used (Fig. 2a; F_1,18_ = 28.5, *p* < *0.001*, ${\eta }_{{\rm{p}}}^{2}$ = 0.61; individual t-test: all *p-values* ≤ *0.017*), with decisive evidence towards an effect of attention on performance (BF_10_ > 100). A repeated-measures 2-way ANCOVA on the changes in performance with attention indicated a significant interaction between emotion and face orientation when controlling for trait anxiety (F_1,18_ = 7.78, *p* = *0.01*2, ${\eta }_{{\rm{p}}}^{2}$ = 0.30), which was supported by substantial evidence (BF_10_ = 4.72). Consistent with the notion that face processing and emotional cues are disrupted for inverted faces^11,14,30^, fearful cues resulted in stronger attentional effects compared to neutral-emotion cues only when presented upright. Upright fearful cues resulted in significantly stronger effects than upright neutral cues (t_19_ = 2.83, *p* = *0.011, d* = *0.63*, BF_10_ = 4.81). When inverted, however, fearful and neutral cues resulted in similar attentional changes (t_19_ = −1.09, *p* = *0.292*, *d* = −*0.24*, BF_10_ = 0.39). In addition, individual trait anxiety (average: 37.3 ± 8.8; range: 24–56) modulated the effects of attention on performance (F_1,18_ = 8.52, *p* = *0.009*, ${\eta }_{{\rm{p}}}^{2}$ = 0.32, BF_10_ = 5.86). The more anxious the participants, the more pronounced the effects of attention on performance (Fig. 2c; r = 0.57, *p* = *0.009*, BF_10_ = 6.6). Changes in performance were due to attentional costs at uncued locations, which became more pronounced with increased anxiety (Fig. 2d; uncued *minus* neutral: r = −0.63, *p* = *0.003*, BF_10_ = 17.3; cued *minus* neutral: r = 0.11, *p* = *0.63*, BF_10_ = 0.31). More important, trait anxiety modulated the interaction of emotion and cue orientation on the attentional changes in performance (F_1,18_ = 6.89, *p* = *0.017*, ${\eta }_{{\rm{p}}}^{2}$ = 0.28, BF_10_ = 3.53), with stronger interaction effects on the attentional changes in high trait anxiety observers (Fig. 2e; r = 0.53, *p* = *0.017*, BF_10_ = 3.93). These results provide further evidence that (i) emotion potentiates the effects of attention on orientation discrimination performance^11,17^, and that (ii) higher trait anxiety is associated with stronger effects of exogenous attention on orientation discrimination performance^10^.

Critically, a similar pattern of results was found for the effects of attention on perceived contrast. Attention enhanced perceived contrast in all conditions (Fig. 2b; F_1,18_ = 60.28, *p* < *0.0001*, ${\eta }_{{\rm{p}}}^{2}$ = 0.77, BF_10_ > 100; individual t-test: all *p-values* < *0.0001*), as indicated by lower and higher PSE estimates when the test and the standard stimuli were cued, respectively. A repeated-measures 2-way ANCOVA on the changes in perceived contrast with attention when controlling for trait anxiety revealed a significant interaction between emotion and face orientation (F_1,18_ = 13.32, *p* = *0.002*, ${\eta }_{{\rm{p}}}^{2}$ = 0.43), although the evidence supporting this interaction was weak (BF_10_ = 1.90). Upright fearful cues resulted in significantly stronger effects on appearance than upright neutral cues (t_19_ = 4.27, *p* < *0.001, d* = *0.96*, BF_10_ = 79.04). When inverted, however, fearful and neutral cues resulted in similar attentional changes (t_19_ = 0.81, *p* = *0.43*, *d* = *0.18*, BF_10_ = 0.31). Individual trait anxiety significantly modulated the overall effects of attention on perceived contrast (F_1,18_ = 11.89, *p* = *0.003*, ${\eta }_{{\rm{p}}}^{2}$ = 0.40, BF_10_ = 11.1), with high trait anxiety observers showing larger attentional enhancement in contrast appearance (Fig. 2f; r = 0.63, *p* = *0.003*, BF_10_ = 17.46). Changes in perceived contrast were reflected for both the test-cued and standard-cued conditions, and modulated by anxiety (Fig. 2g; *standard-cued minus neutrally-cued*: r = 0.63, *p* = *0.003*, BF_10_ = 17.91; *test-cued minus neutrally-cued*: r = −0.61, *p* = *0.004*, BF_10_ = 12.02). Moreover, there was substantial evidence that trait anxiety modulated the interaction of emotional valence and face orientation (F_1,18_ = 6.49, *p* = *0.020*, ${\eta }_{{\rm{p}}}^{2}$ = 0.27, BF_10_ = 3.14), with high trait-anxiety observers showing a stronger preferential enhancement in perceived contrast with upright fearful face cues (Fig. 2h; r = 0.51, *p* = *0.020*, BF_10_ = 3.44). These results reveal that both emotional cues and internal states related to trait anxiety strongly modulate the way exogenous attention alters our subjective experience.

**Figure 3:**
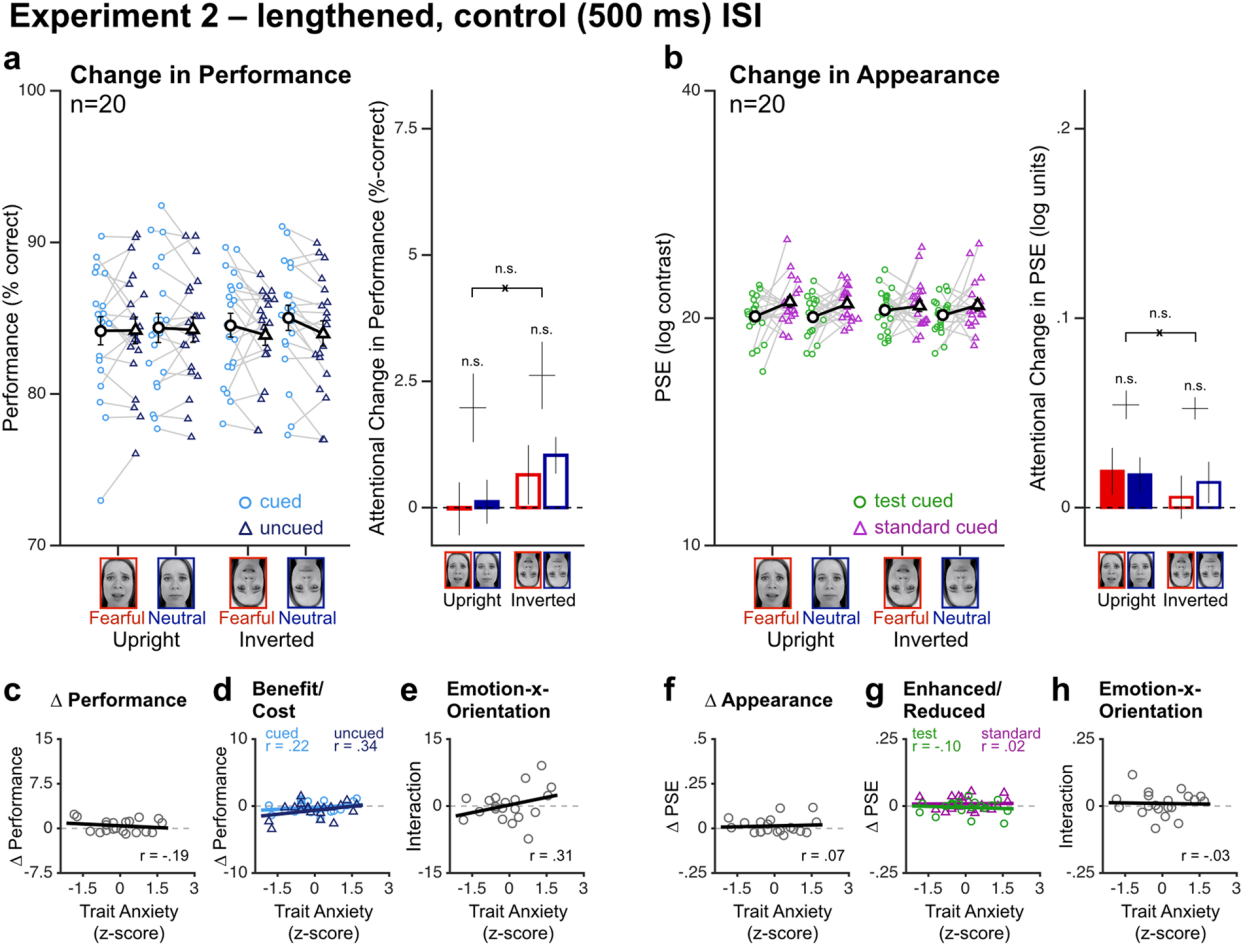
Experiment 2 (lengthened ISI, control). (**a**,**b**) No attentional change on either (**a**) orientation discrimination performance or (**b**) perceived contrast was observed when the ISI between the cue and the stimulus was lengthened, consistent with the transient nature of the effects of exogenous attention. (**c**–**h**) Individual trait anxiety had no influence on the magnitude of the attentional effects or the interaction between attention and emotion. Same conventions as Fig. 2.

To ensure that these results reflected the effects of exogenous attention, which peak around 100–120 ms after cue onset^1^, we conducted a control experiment (Experiment 2; n = 20) using the exact same procedure as in Experiment 1 except that a longer ISI (500 ms) was used between cue offset and stimulus onset (Fig. 1a). Given the transient nature of exogenous attention^1^, lengthening the ISI by a few hundred milliseconds is enough to abolish its effects^23–25^. As expected, we found that the effects of the attentional precues vanished for both performance (Fig. 3a) and perceived contrast (Fig. 3b) when the ISI between the cue and stimuli was lengthened to 500 ms. Cueing did not alter performance (F_1,18_ = 3.58, *p* = *0.075*, η_p_^2^ = 0.17, BF_10_ = 0.85) or perceived contrast (F_1,18_ = 2.03, *p* = *0.171*, η_p_^2^ = 0.10; although there was evidence suggesting a small main effect of attention BF_10_ = 15.9). Critically, emotion and face orientation did not interact (performance: F_1,18_ = 0.10, *p* = *0.76*, η_p_^2^ = 0.01, BF_10_ = 0.31; perceived contrast: F_1,18_ = 0.89, *p* = *0.359*, η_p_^2^ = 0.05, BF_10_ = 0.41). Moreover, trait anxiety (average: 38.2 ± 7.6; range: 24–51) had no influence on either performance (Fig. 3c–e; main effect: F_1,18_ = 0.69, *p* = *0.42*, η_p_^2^ = 0.04, BF_10_ = 0.36; emotion-x-orientation interaction: F_1,18_ = 1.94, *p* = *0.180*, η_p_^2^ = 0.10, BF_10_ = 0.77) or perceived contrast (Fig. 3f–h; main effect: F_1,18_ = 0.10, *p* = *0.76*, η_p_^2^ = 0.01, BF_10_ = 0.48; emotion-x-orientation interaction: F_1,18_ = 0.02, *p* = *0.90*, η_p_^2^ = 0.001, BF_10_ = 0.40). Thus, unlike in Experiment 1 where the ISI was optimal for exogenous attention, lengthening the ISI abolished the effects of attention, as well as the influence of emotion-relevant modulatory signals and individual trait anxiety, on both orientation discrimination and perceived contrast.

**Figure 4:**
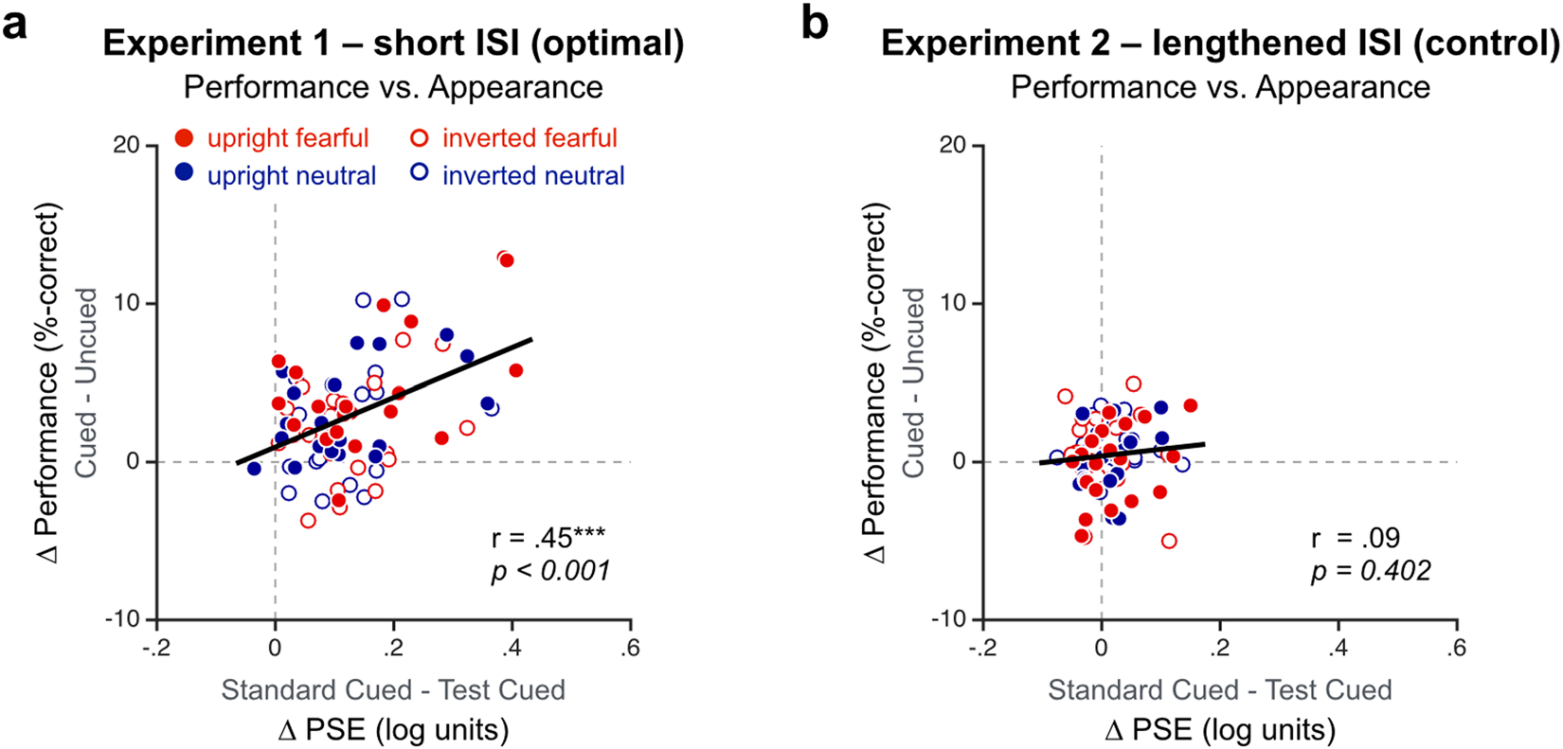
The effects of attention on performance and on appearance correlated with each other (**a**) in Experiment 1 (short, optimal ISI for exogenous attention), but not (**b**) in Experiment 2 (control, lengthened ISI). Each dot represents individual attentional changes for each of the four different types of attentional precues.

Finally, we tested the notion that the attentional changes in performance and contrast appearance are linked to each other, and may reflect a common attention mechanism. Consistent with this idea, the magnitude of the effects of attention on orientation discrimination performance and contrast appearance correlated with each other in Experiment 1, when using either each of the 4 different cue conditions for each observer (Fig. 4a**;** r = 0.45, *p* < *0.0001*, BF_10_ > 100) or the overall attentional change for each observer (r = 0.54, *p* = *0.013*, BF_10_ = 4.82). No such correlation was found in Experiment 2 with the lengthened ISI (Fig. 4b**;** r = 0.095; *p* = *0.402*, BF_10_ = 0.20; using overall changes: r = 0.11; *p* = *0.63*, BF_10_ = 0.31).

## Discussion

Exogenous attention represents an adaptive tool from an evolutionary perspective, allowing us to rapidly and automatically detect salient events in our environment. Here, we demonstrated that: (i) emotional cues potentiate the effects of attention on both performance and contrast appearance; (ii) individual trait anxiety mediates both the effects of exogenous attention as well as the interactions between emotion and attention; and (iii) the individual effects of attention on performance and appearance correlated with each other, consistent with the notion that these effects may reflect a common attentional mechanism. Altogether, these findings show for the first time that both the emotional content of visual information and our own internal anxiety state influence attention in a way that truly alters how visual information appears to us.

Our results further illustrate the fact that exogenous attention benefits performance in orientation discrimination tasks^1,3^, but also enhances contrast appearance^23–29^. More important, our results reveal that emotional cues potentiate the effects of exogenous attention not only on perceptual performance^9–11,15,17^, but also on visual appearance. Relative to non-emotional cues, fearful face cues resulted in larger attentional benefits in orientation discrimination performance, as well as in larger enhancement in perceived contrast. Consistent with the fact that both emotional and face processing are disrupted for inverted faces^11,14,30^, this emotional potentiation of attention was observed only when comparing upright–but not inverted–cues, ruling out low-level differences between fearful and neutral face cues as a possible explanation.

Exogenous attention is a reflexive mechanism that benefits performance in many visual tasks^1,3,4^. Hence, the influence of emotional signals on attentional selection confers a perceptual advantage in most circumstances^9–11,15,17^. However, exogenous attention is inflexible and automatically affects visual processing, even when detrimental for the task at hand^1,4,15,32–34^. For instance, exogenous attention increases spatial resolution and reduces temporal resolution, which can actually impair performance when either lower resolution^4,32,33^ or faster temporal resolution^15,34^ is required. Fearful attentional cues potentiate this trade-off in spatiotemporal resolution^15^, resulting in both stronger spatial resolution benefits and temporal resolution costs. Thus, emotional signals in our environment are particularly salient cues that automatically capture exogenous attention towards the cued location and affect how we see regardless of the task at hand.

Several studies have begun to uncover the neural mechanisms underlying the interaction of emotion and attention on visual processing. Activation of visual cortical areas by emotionally relevant information is assumed to be due to feedback projections from the amygdala to cortical visual areas, including V1^12^. Fearful faces like the ones we used as cues lead to stronger activations of ^5,7,8,17^, and correlated activity between^13^ the amygdala and visual processing areas. Amygdala damage in humans^35^ or inactivation in monkeys^36^ result in a lack of emotion-related enhancement of visual areas in response to fearful faces. Importantly, the amygdala has connections to other cortical regions^5,7,8,19^–such as the orbitofrontal cortex, DLPFC, and posterior parietal cortex–which are involved in the control and orienting of attention^5–7,17^. For instance, the orbitofrontal cortex mediates the interaction between emotion and attention, biasing attention towards emotional images^37^. Moreover, feedback from the DLPFC to V1 may mediate the interaction between emotional valence and the attentional changes in contrast sensitivity occurring in V1^17^. The effects of emotional stimuli seem to be mediated by the low spatial frequency content^16,38^, which has been shown to be rapidly transmitted to both the amygdala^12^ and the orbitofrontal cortex^39^. Thus, response to a fearful face could rapidly modulate brain areas involved in attentional selection and direct stimulus-driven attention towards locations of possible threat, potentiating the effects of exogenous attention and altering our perception.

Our findings also provide evidence that internal arousal states related to trait anxiety can intrinsically impact the way emotion and attention affect perception. Consistent with the notion that internal states play a major role in regulating brain functions, we found that trait anxiety had a pronounced modulatory effect on exogenous attention. Anxiety embodies a state of high arousal and enhanced vigilance in the absence of an immediate threat, which can aid survival by enabling rapid responses to possible hazards. For instance, threat stimuli can capture and hold spatial attention more strongly than neutral stimuli, particularly in anxious individuals, resulting in slower disengagement from potential threat stimuli^7,9,10,21,22^. Our results provide additional evidence that threat stimuli are associated with delayed disengagement rather than enhanced capture in anxious observers. Physiologically, trait anxiety has been associated with a hyper-responsive threat-detection system centered on the amygdala and with reduced recruitment of prefrontal attentional control mechanisms^19^. High-anxiety individuals exhibit heightened amygdala activity in response to potential threat^40^, which could in turn bias the allocation of attention towards locations of threat-related stimuli. Anxiety is also associated with a reduced activation of prefrontal regions involved in attentional control and distractor suppression^18,20^. The imbalance between stimulus-driven emotional response and top-down attention control may underlie anxious individuals’ difficulty with disengaging attention from threat-related stimuli, explaining why trait anxiety results in stronger exogenous attention effects and in stronger interaction effects between emotion and exogenous attention. Future studies should investigate whether the deregulation of attentional control mechanisms in anxious observers impacts the effects of endogenous (voluntary, goal-driven) attention, which has also been shown to improve performance^1,3,4,41^ and alter visual appearance^42–44^ in various tasks.

Another important and novel finding is the correlation between the changes in performance and the changes in perceived contrast brought about by exogenous attention. Although such correlation does not necessarily indicate a joint origin of these effects, it is consistent with the hypothesis that both changes reflect a common attentional mechanism modulating contrast sensitivity responses in early sensory cortex^23,27–29,45–47^. Many neurophysiological studies have demonstrated that the attentional mechanisms mediating changes in contrast sensitivity and orientation discrimination performance take place in early visual cortex^1–3,28,48^. Links between the physiological and perceptual effects of attention on contrast perception have been suggested^2,27–29,45–47^. Some neurophysiological studies have shown that attention affects neuronal activity via a shift of the contrast-response function towards lower contrasts^46,49^, consistent with the idea that attention enhances the effective contrast of a stimulus. Such neural enhancement in effective contrast with attention is likely correlated with an increase in contrast sensitivity and could explain enhanced contrast appearance with attention. Consistent with this idea, an EEG study^28^ showed that modulations of evoked potentials in contralateral occipital cortex correlated with the behavioral report of enhanced perceived contrast. Our findings provide additional support to converging neurophysiological and behavioral evidence^2,27–29,45–47^ that the changes in contrast sensitivity and perceived contrast with attention are both likely due to the attentional boost of early sensory processing. Thus, attention is a mechanism that can provide an optimized representation of visual information around us, enhancing relevant details at the expense of a faithful representation of the sensory input.

It has been proposed that response bias^50^ may explain the effects of exogenous attention on appearance. In addition to many control experiments in previous studies^23–26,42–44^, several of our present findings rule out this possibility. First, as in previous control experiments^23,25^, lengthening the ISI (Experiment 2) abolished the effects observed with the optimal ISI for exogenous attention (Experiment 1), which is consistent with the transient dynamics of exogenous attention^1^. The lack of cueing effects in the lengthened ISI condition also indicates that inhibition of return^51^ did not occur in our task at this delay, as it would have resulted in impaired performance at the cued location. Second, the correlation we observed between the effects of exogenous attention on performance and appearance may reflect a common mechanism^27^. Moreover, the interaction of emotional valence (fearful vs neutral-emotion) and face orientation (upright vs inverted) on the effects of attention on appearance, as well as the influences of trait anxiety, further rule out low-level visual effects, response bias, or cognitive strategies as possible explanations. These findings strongly support that changes in appearance are a perceptual consequence of the attentional boost observed in early sensory processing.

The present study provides novel evidence that both emotion and anxiety influence the way attention alters performance and visual appearance at early stages of visual processing, illustrating the notion that brain functions depend on distributed networks with highly interactive dynamics. Altogether, our results reveal that by potentiating the effects of exogenous attention on both performance and appearance, emotion and anxiety can truly alter how we see.

## Methods

### Participants

Twenty observers participated in each experiment (Exp. 1: 13 females, mean age 23.9 ± 5.04 yo, range: 19–38; Exp. 2: 14 females, mean age 24.4 ± 5.8 yo, range: 19–40). All were naive as to the purposes of the experiment and had normal or corrected-to-normal vision. In each experiment, about half were inexperienced psychophysical observers. The Institutional Review Board at New York University approved the experimental procedures and all participants gave informed consent. All experiments were performed in accordance with the Declaration of Helsinki.

### Apparatus

Stimuli were generated using Matlab (MathWorks, Natick, MA) with the Psychtoolbox extensions^52^ and were displayed on a 22′′ CRT monitor (1280 × 960 at 100-Hz) situated at 57-cm. Background luminance was set 57 cd/m^2^. The display was calibrated using a Photo Research (Chatworth, CA) PR650 SpectraColorimeter to produce linearized lookup tables.

### Stimuli

Two oriented Gabor stimuli (2 cycle/deg; sigma of the Gaussian envelope: 0.85 deg) were presented at isoeccentric locations to the left and right (±8 deg) of a central fixation dot, 1 deg° under the horizontal meridian. One stimulus had a reference contrast (20%; standard stimulus) while the other varied in contrast in log-space (6.3–63%; test stimulus). Stimulus orientation was set for each participant during the initial training session and varied across each block to maintain performance near 80% correct (Exp. 1–average tilt: 2.2° ± 0.7°, range: 1.3°–3.7°; Exp. 2–average tilt: 2.7° ± 1°, range: 1.5°–5.1°). Spatial attention was manipulated using non-informative precues corresponding to upright or inverted Ekman faces (5.25 × 7 deg) with either fearful or neutral emotion expressions (Fig. 1a). Such non-informative precues have been successfully used to manipulate exogenous attention in previous studies^10,11,15^. Attentional precues were presented ± 8 deg to the left and right of the central fixation, and centered 7.5 deg above the horizontal meridian to avoid spatial overlap with stimulus locations. We sampled from the Pictures of Facial Affect series^53^ prototypical exemplars of fearful and neutral expressions. The set included 11 unique exemplars of each emotion category (6 female faces and 5 male faces), for a total of 22 unique facial stimuli. Each fearful expression had a neutral counterpart posed by the same person. These faces had been used by Phelps, Ling and Carrasco^11^, who had verified that observers could report with greater than 90% accuracy the facial expression of both expression types using similar size, eccentricity, and timing parameters. To rule out the possibility that differential effects of fearful and neutral face cues could be due to low-level characteristics, inverted fearful and neutral face cues were also used (control condition), such that their physical content remained the same but their emotional content was not readily processed^11,14,30^.

### Procedure

In each trial, observers saw two oriented Gabor stimuli presented simultaneously to the left and right of a central fixation (Fig. 1a). Following a 500 ms fixation window, either one or two non-informative peripheral precues appeared for 80 ms at either one (focal attention) or both (distributed attention) locations. In the distributed attention condition, the two precues were identical. Two Gabor stimuli were presented for 40 ms, following either a short 40 ms (Exp. 1–optimal for exogenous attention) or a lengthened 500 ms (Exp. 2–control condition) inter-stimulus interval (ISI). Observers were asked to report the orientation (CCW or CW) of the higher contrast stimulus, allowing us to measure both orientation discrimination performance and contrast appearance with one single button press (left stimulus/hand: ‘Z’:CCW, ‘X’:CW; right stimulus/hand: ‘<’:CCW, ‘>’:CW;). No feedback was provided. Trait anxiety was assessed for each participant using the State-Trait Anxiety Inventory (STAI)^31^. Each participant ran a total of 5184 trials (4 face cue types x 3 cueing conditions x 9 contrast levels: 48 trials per data point) divided across 4–6 separate sessions. Observers were under no time pressure to respond, but trials for which reaction times were longer than 3 s were excluded from the analysis (Exp. 1: 0.48% ± 0.58%, range: 0.02–2.5%; Exp. 2: 0.75% ± 0.97%, range: 0.02–3.6%).

### Performance and appearance measures

The effects of attention on orientation discrimination performance (Fig. 1b) were computed for each of the four precue conditions by measuring the differences in performance when the reported stimulus was cued minus when the reported stimulus was uncued. For the appearance judgment, we computed the percentage of trials in which the test stimulus was reported as higher contrast as a function of test log contrast (Fig. 1c). Data for each cueing condition (test-stimulus cued, standard-stimulus cued, neutral-distributed condition) and each cue type (upright or inverted fearful faces; upright or inverted neutral faces) were then fitted with a Weibull function:

1 $$\psi =\gamma +(1-\gamma -\lambda )\{1-exp[-{(\frac{x}{\alpha })}^{\beta }]\}$$

in which *Ψ* is the proportion of trials the test stimulus was chosen as higher contrast over the standard stimulus; *x* is the contrast of the test stimulus; *α* is the location parameter; *β* is the slope; and *γ* and *λ* are the lower and upper asymptotes respectively, using maximum likelihood procedure. The points of subjective equality (PSE) were derived from individual fits for each condition as the test contrast at which the test and standard stimuli are subjectively indistinguishable and equally likely to be chosen as higher contrast. The effects of attention on appearance were computed by taking the difference in PSEs when attention was at the test-stimulus location minus when attention was at the standard-stimulus location (Fig. 1c).

### Statistical analyses

All statistical tests were conducted using both SPSS and JASP for frequentist analyses, and JASP for Bayesian analyses. Repeated-measures analysis of covariance (ANCOVAs) with trait anxiety as a covariate were used to assess the effects of emotional valence (fearful or neutral), cue orientation (upright or inverted) on the effects of attention on orientation discrimination performance (cued *minus* uncued) and on perceived contrast (standard cued PSE *minus* test cued PSE). In all cases in which the Mauchly’s test of sphericity indicated a violation of the sphericity assumption, the Greenhouse-Geisser corrected values were used. Partial eta-squared (${\eta }_{p}^{2}$) and Cohen’s *d* values were calculated to assess effect size for ANCOVAs and paired sample t-tests. Trait-anxiety scores from the STAI were standardized (i.e., z-score) and correlated to the effects of attention on appearance and on performance using Pearson’s correlation. Normality was assessed using the Royston’s test for multivariate normality^54^ and the Mahalanobis distance was computed to detect the presence of outlier participants (with alpha values at 0.1). Performance changes as a function of observer’s trait anxiety were normally distributed for all conditions (all *p-values* > *0.1*) and no outlier was found. Changes in perceived contrast were normally distributed in 3 out of 4 conditions (upright-fearful: p = 0.13; upright-neutral: p = 0.19; inverted-fearful: p = 0.54; inverted-neutral: p = 0.034). ANCOVAs are quite robust to such small deviations from normality, which was due to the presence of one outlier (no deviation from normality was observed after excluding this participant; all *p-values* > *0.2*). Excluding this observer from the analysis did not affect our findings (e.g., emotion-x-orientation interaction on appearance changes: F_1,17_ = 10.45, *p* = *0.005*, ${\eta }_{{\rm{p}}}^{2}$ = 0.38). Moreover, similar results were found using a non-parametric analysis (adjusted ranked test^55^: emotion-x-orientation interaction on appearance changes F_1,18_ = 9.59, *p* = *0.006*, ${\eta }_{{\rm{p}}}^{2}$ = 0.33).

Bayesian analyses^56–58^ were conducted to further characterize our effects. Contrary to classical frequentist approaches, Bayesian analyses can provide evidence in favor of significant effects as well as in favor of null effects by comparing different models (e.g., with main effects and/or interactions of interest) with the null model. This method can thus provide evidence in favor of the null hypothesis (i.e., by showing that the data are better explained by the null model than any other models). Bayesian ANCOVAs, correlations, and t-tests were used to generate Bayes Factors (BF_10_), which represent the evidence in favor of a model of interest relative to the null model. A BF_10_ smaller than 1 represents evidence in favor of the null model. A BF_10_ greater than 1 indicates that the model of interest performs better than the null model, with supporting evidence being considered either weak/anecdotal (1 < BF_10_ < 3), substantial (3 < BF_10_ < 10), strong (10 < BF_10_ < 30), very strong (30 < BF_10_ < 100) or decisive (BF_10_ > 100). Evidence in favor of the null model can be qualified as either weak/anecdotal (0.33 < BF_10_ < 1), substantial (0.1 < BF_10_ < 0.33), strong (0.033 < BF_10_ < 0.1), very strong (0.01 < BF_10_ < 0.033) or decisive (BF_10_ < 0.01).

### Data availability

All data are available from the authors upon request.
